# Potential insect threats to pennycress, *Thlaspi arvense* (Brassicales: Brassicaceae), an emerging oilseed cover crop

**DOI:** 10.1093/jisesa/ieae086

**Published:** 2024-08-27

**Authors:** Ellen O Adjeiwaa, Arthur V Ribeiro, Robert L Koch

**Affiliations:** Department of Entomology, University of Minnesota, 1980 Folwell Avenue, Saint Paul, MN 55108, USA; Department of Entomology, University of Minnesota, 1980 Folwell Avenue, Saint Paul, MN 55108, USA; Department of Entomology, University of Minnesota, 1980 Folwell Avenue, Saint Paul, MN 55108, USA

**Keywords:** Brassicaceae, herbivores, pest, pennycress

## Abstract

Pennycress (*Thlaspi arvense* L.) is an annual plant in temperate regions that often grows as a weed. Pennycress is being domesticated as a new winter cover crop and oilseed crop for incorporation in the Midwest United States corn-soybean rotation, where it could offer economic and environmental benefits. While pennycress is gaining attention as a promising new crop, there remains a significant gap in understanding its interaction with insect communities and agroecosystems. This review compiles available information on insect herbivores (potential pests) and beneficial insects associated with pennycress growing in the wild (natural areas) or as a weed in agricultural areas. The limited knowledge on the response of pennycress to stressors (defoliation, stem injury and stand loss) similar to injury that could be caused by insects is also compiled here. By shedding light on the insects associated with pennycress and how pennycress might respond to injury from insect pests, this review sets the stage for further research and development of integrated pest management programs for insect pests of this new crop.

## Introduction

Pennycress (*Thlaspi arvense* L. (Brassicales: Brassicaceae)) is native to Eurasia and is now found in Europe, Asia, North America, South America, Africa, and Australia (Best and McIntyre 1975, Warwick et al. 2002). In the United States, pennycress was first documented in 1818 (Mitich 1996), and it has been a problematic weed for multiple crops (Warwick et al. 2002).

Pennycress is being considered as a new oil seed crop and winter cover crop (Sedbrook et al. 2014). The oil content in pennycress varies between 26% and 39% of the seed weight (Cubins et al. 2019). Pennycress meal is a beneficial source of protein as pennycress seeds have protein content ranging between 20% and 27% of the seed weight (Hojilla-Evangelista et al. 2013, Selling et al. 2013). Furthermore, pennycress is a desirable low-input winter cover crop with the potential to reduce soil erosion, take up excess nitrogen from the soil, and contribute to weed suppression (Evangelista et al. 2017, Cubins et al. 2019, Moore et al. 2020). In the Midwest United States, pennycress would likely be incorporated into the cropping systems (e.g., corn-soybean rotations) through double- or relay-cropping. For example, pennycress would be planted after the harvest of corn or possibly interseeded with the corn prior to the harvest of the corn (Cubins et al. 2019, Mohammed et al. 2020, Moore et al. 2020). The pennycress crop would overwinter, and soybean would be relay cropped into the pennycress prior to pennycress harvest or double-cropped after pennycress harvest (Johnson et al. 2017, Cubins et al. 2019, Cecchin et al. 2021).

Domestication of pennycress is advancing rapidly due to the availability of a diversity of pennycress genotypes and the sequenced genome and transcriptome of this plant (Sedbrook et al. 2014, Basnet and Ellison 2024). Pennycress seeds contain glucosinolates, primarily sinigrin, which provide protection against herbivores but can adversely affect some end uses for pennycress meal. Therefore, efforts are underway to lower the glucosinolate levels in pennycress seeds (Vaughn et al. 2005, Chopra et al. 2020). Domestication of crops in the past has led to increased vulnerability to insect pests (Chen and Welter 2005). The domestication of pennycress is changing its chemical defenses, which may impact its vulnerability to insect herbivores (e.g., Chen et al. 2018). The effects of pennycress domestication on insect herbivores remain unknown.

While there have been significant efforts to improve the agronomics (e.g., planting, fertilizing, and harvesting) of pennycress as a new crop (Cubins et al. 2019), there has been little focus on studying the potential insect pests that could affect this crop. Experience with related crops, such as canola and rapeseed (*Brassica napus* L. (Brassicales: Brassicaceae)) suggests that insect pests could pose considerable threats to pennycress production. A diverse community of insect pests can attack various parts of canola and rapeseed plants, including the roots, stems, leaves, flowers, seed pods, and seeds (Lamb 1989, Alford et al. 2003, Williams 2010, Zheng et al. 2020). From a worldwide perspective, insect pests that are problematic to these crops on multiple continents include diamondback moth (*Plutella xylostella* (L.) (Lepidoptera: Plutellidae)), aphids (Hemiptera: Aphididae), flea beetles (*Phyllotreta* spp. (Coleoptera: Chrysomelidae)), cabbage root fly (*Delia radicum* (L.) (Diptera: Anthomyiidae)), cabbage seedpod weevil (*Ceutorhynchus obstrictus* (Marsham) (Coleoptera: Curculionidae)), cutworms (Lepidoptera: Noctudidae), wireworms (Coleoptera: Elateridae), pollen beetle (*Brassicogethes aeneus* (F.) (Coleoptera: Nitidulidae)), and brassica pod midge (*Dasineura brassicae* (Winnertz) (Diptera: Cecidomyiidae)) (Zheng et al. 2020). Chemical control remains the predominant tactic for the management of insect pests on these crops (Lamb 1989, Alford et al. 2003, Williams 2010, Zheng et al. 2020), though advances are being made in biological control, cultural control, and host-plant resistance (Lamb 1989, Alford et al. 2003, Williams 2010, Zheng et al. 2020).

Here, we provide a review of insects that have been reported in association with pennycress and how pennycress responds to stressors similar to the feeding injury that could be caused by some insects. This information will help to provide a foundation for the development of integrated pest management programs for this new crop. Current species names, as determined by searching the Integrated Taxonomic Information System and the National Center for Biotechnology Information (NCBI) Taxonomy Database, are listed in parentheses following the species names used in the literature.

## Herbivorous Insects Associated with Pennycress

A comprehensive review of the literature found reports of herbivorous insects from 9 countries associated with pennycress (Table 1). A total of 119 species of herbivorous insects across 6 insect orders were reported in association with above- and below-ground parts of pennycress plants (Table 1). Various life stages of these species have been reported with pennycress (Table 1). For the insects that have been studied more thoroughly, a review of their interactions with pennycress is provided below. In addition to insects, two-spotted spider mite (*Tetranychus urticae* (Koch) (Trombidiformes: Tetranychidae)), which is known as a pest of many crops, was also found on pennycress (Lipa et al. 1977).

**Table 1.** Insect herbivores reported in association with pennycress (*Thlaspi arvense*)

Order	Family	Species^a^	Country^b^	Life stage^c^	Plant part^d^	Reference
Coleoptera	Cantharidae	*Cantharis lateralis*	Poland	A	S, L	Lipa et al. 1977
	Chysomelidae	*Chaetocnema concinna*	Poland	A	S, L	Lipa et al. 1977
		*Gastroidea polygoni*	Poland	A	S	Lipa et al. 1977
		*Haltica palustris*	Poland	A	S	Lipa et al. 1977
		*Phyllotreta armoraciae*	Denmark	–	L	Nielsen et al. 1979
		*Phyllotreta atra*	Poland & Czech Republic	A	S, L	Lipa et al. 1977, Štolcová 2009
		*Phyllotreta cruciferae*	Poland, Denmark, & Canada	A	S, L	Lipa et al. 1977, Gavloski et al. 2000, Palaniswamy et al. 1997, Nielsen et al. 2001
		*Phyllotreta flexuosa*	Poland	A	S, L	Lipa et al. 1977, Nielsen et al. 2001
		*Phyllotreta nemorum*	Poland & Denmark	A	S, L	Lipa et al. 1977, Nielsen et al. 2001
		*Phyllotreta nigripes*	Czech Republic	–	L	Štolcová 2009
		*Phyllotreta striolata*	Canada	–	–	Meisner and Mitchell 1983
		*Phyllotreta undulata*	Poland	A	S, L	Lipa et al. 1977
		*Phyllotreta vittula*	Poland	A	S, L	Lipa et al. 1977
		*Psylliodes napi*	Poland	A	S, L	Lipa et al. 1977
	Curculionidae	*Ceutorhynchus assimilis*	Poland	A	S, L	Lipa et al. 1977
		*Ceutorhynchus chalybaeus*	Poland	A	S, R	Lipa et al. 1977
		*Ceutorhynchus contractus*	Poland	A	L	Lipa et al. 1977
		*Ceutorhynchus erysimi*	Poland	A	S, R	Lipa et al. 1977
		*Ceutorhynchus floralis*	Poland	A	S, L, SP	Lipa et al. 1977
		*Ceutorhynchus granulicollis*	Poland	A	S, L	Lipa et al. 1977
		*Ceutorhynchus napi*	Poland	A	S, L	Lipa et al. 1977
		*Ceutorhynchus obstrictus*	Canada	A	L	Dosdall and Moisey 2004
		*Ceutorhynchus pleurostigma*	Poland	A	S, L	Lipa et al. 1977
		*Ceutorhynchus pulvinatus*	Poland	A	S, L, SP	Lipa et al. 1977
		*Ceutorhynchus quadridens*	Poland	A	S, R	Lipa et al. 1977
		*Ceutorhynchus roberti*	Poland	A	S, L	Lipa et al. 1977
		*Hypera postica*	United States	A, E	S	Saad and Bishop 1969
		*Trachyphloeus bifoveolatus* (*Romualdius bifoveolatus*)	Poland	A	O	Lipa et al. 1977
	Dermestidae	*Anthrenus pimpinellae*	Poland	A	O	Lipa et al. 1977
	Elateridae	*Agriotes lineatus*	Poland	A	O	Lipa et al. 1977
		*Athous niger*	Poland	L	R	Lipa et al. 1977
	Melyridae	*Malachius marginellus*	Poland	A	S	Lipa et al. 1977
	Nitidulidae	*Meligethes aeneus* (*Brassicogethes aeneus*)	Poland	A	F	Lipa et al. 1977
		*Meligethes lugubris* (*Thymogethes lugubris*)	Poland	A	O	Lipa et al. 1977
		*Meligethes picipes*	Poland	A	O	Lipa et al. 1977
		*Meligethes planiusculus*	Poland	A	O	Lipa et al. 1977
		*Olibrus aeneus*	Poland	A	O	Lipa et al. 1977
	Phalacridae	*Olibrus corticalis*	Poland	A	O	Lipa et al. 1977
		*Stilbus testaceus*	Poland	A	O	Lipa et al. 1977
Diptera	Agromyzidae	*Liriomyza brassicae*	United States	A, E, L	L	Tavormina 1982
		*Liriomyza strigata*	Poland	A	L	Lipa et al. 1977
		*Phytomyza horticola*	Poland	A	L	Lipa et al. 1977
		*Phytomyza rufipes*	Poland	A	L	Lipa et al. 1977
	Anthomyiidae	*Delia floralis*	Canada	–	–	Strickland 1938 Savage et al. 2016
		*Delia platura*	United States	A	F	Forcella et al. 2023a
		*Erioischia brassicae* (*Delia radicum*)	United Kingdom	A, E, L	–	Finch and Ackley 1977
	Cecidomyiidae	*Contarinia nasturtii*	Poland & United States	A, E, L	SP, L	Lipa et al. 1977 Chen et al. 2009
	Drosophilidae	*Scaptomyza flavoela*	Poland	A, L, P	L	Lipa et al. 1977
Hemiptera	Aphididae	*Acyrthosiphon pisum*	Poland	N^e^, A	S, L	Lipa et al. 1977
		*Aphis craccivora*	Poland	A, N^e^	S, L	Lipa et al. 1977
		*Aphis fabae*	Poland	A, N^e^	S, L	Lipa et al. 1977
		*Aphis nasturtii*	Poland	A, N^e^	S, L	Lipa et al. 1977
		*Aulacorthum solani*	Poland	N^e^	S, L	Lipa et al. 1977
		*Brevicoryne brassicae*	Poland	A, N	S, L	Gabryś and Pawluk 1999
		*Cavariella aegopodii*	Poland	A, N^e^	O	Lipa et al. 1977
		*Hyadaphis pseudobrassicae* (*Lipaphis pseudobrassicae*)	United States	A	–	Jarvis 1970
		*Lipaphis erysimi*	Poland	A, N^e^	S, L	Lipa et al. 1977
		*Lipaphis* sp.	Poland	N^e^	S, L	Lipa et al. 1977
		*Myzus cerasi*	Poland	A, N^e^	O	Lipa et al. 1977
		*Myzodes persicae (Myzus persicae)*	Poland	A, N	S, L	Lipa et al. 1977
		*Rhopalosiphum padi*	Poland	A	O	Lipa et al. 1977
		*Sitobion* sp.	Poland	N^e^	O	Lipa et al. 1977
	Aphrophoridae	*Philaenus spumarius*	Poland	A, L	O	Lipa et al. 1977
	Cicadellidae	*Athysanus quadrum*	Poland	A	O	Lipa et al. 1977
		*Eupteryx atropunctata*	Poland	A	O	Lipa et al. 1977
		*Euscelidius schenckii*	Poland	A, N^e^	O	Lipa et al. 1977
		*Macrosteles cristatus*	Poland	A	O	Lipa et al. 1977
		*Macrosteles laevis*	Poland	A	O	Lipa et al. 1977
		*Macrosteles sexnotatus*	Poland	A	O	Lipa et al. 1977
		*Macrosteles* sp.	Poland	A	O	Lipa et al. 1977
		*Psammotettix alienus*	Poland	A	O	Lipa et al. 1977
		*Psammotettix* sp.	Poland	A	O	Lipa et al. 1977
	Delphacidae	*Javesella pellucida*	Poland	A	O	Lipa et al. 1977
		*Javesella* sp.	Poland	A	O	Lipa et al. 1977
		*Laodelphax striatella* (*Laodelphax striatellus*)	Poland	A	O	Lipa et al. 1977
	Lygaeidae	*Cymus claviculus*	Poland	A	O	Lipa et al. 1977
		*Nysius ericae*	Poland	A	O	Lipa et al. 1977
		*Nysius senecionis*	Poland	A	O	Lipa et al. 1977
	Miridae	*Adelphocoris lineolatus*	Poland	A	O	Lipa et al. 1977
		*Apolygus limbatus*	Poland	A	O	Lipa et al. 1977
		*Calocoris norvegicus*	Poland	A	O	Lipa et al. 1977
		*Lygus borealis*	Canada	A	–	Gerber and Wise 1995
		*Lygus elisus*	Canada	A		Gerber and Wise 1995
		*Lygus gemellatus*	Poland	A	S, L	Lipa et al. 1977
		*Lygus lineolaris*	Canada	A, N	–	Gerber and Wise 1995
		*Lygus pratensis*	Poland	A	S, L	Lipa et al. 1977
		*Lygus rugulipennis*	Poland	A	S, L	Lipa et al. 1977
		*Lygus* sp.	Poland	N^e^	S, L	Lipa et al. 1977
		*Orthotylus flavosparsus*	Poland	A	R	Lipa et al. 1977
		*Polymerus vulneratus*	Poland	A	S, L	Lipa et al. 1977
	Pentatomidae	*Carpocoris fuscispinus*	Poland	A, E	–	Modnicki et al. 2019
		*Dolycoris baccarum*	Poland	A, E	–	Modnicki et al. 2019
		*Eurydema oleracea*	Poland	A, N	S, L	Lipa et al. 1977
		*Eurydema ornata*	Poland	A	S, L	Lipa et al. 1977
	Triozidae	*Bactericera nigricornis*	Poland	A, N	S, L	Lipa et al. 1977
Hymenoptera	Tenthredinidae	*Athalia colibri*	Poland	A	O	Lipa et al. 1977
Lepidoptera	Noctuidae	*Prodenia eridania* (*Spodoptera eridania*)	United States	L	L	Soo Hoo and Fraenkel 1966
		*Trichoplusia ni*	Canada	L	L	Cameron et al. 2007
	Pieridae	*Leptidea sinapis*	Poland	P	S, L	Lipa et al. 1977
		*Pieris brassicae*	Poland	A, L	S, L	Lipa et al. 1977
		*Pieris napi*	Poland, United States, Sweden, & Japan	A, E, L	S, L	Lipa et al. 1977, Forsberg 1987
		*Pieris napi macdunnoughii* (*Pieris macdunnoughi*)	Poland, United States, & Sweden	A, E, L	S, L	Chew 1975, 1977, 1980, Lipa et al. 1977, Nakajima et al. 2013
		*Pieris occidentalis*	United States	A, L	L	Chew 1975, 1980, 1977
		*Pieris rapae*	Poland, United States, & Japan	E, L, P	S, L	Okamura et al. 2016, Chew 1975, Slansky and Feeny 1977
	Plutellidae	*Plutella maculipennis* (*Plutella xylostella*)	China, United States, Canada & Poland	A, E, L, P	S, L	Niu et al. 2014, Harcourt 1957, Idris and Grafius 1996, Kmec and Weiss 1997, Munir et al. 2018, Lipa et al. 1977
	Saturniidae	*Samia cynthia ricini* (*Samia ricini*)	Japan	L	–	Okamura et al. 2016
	Tortricidae	*Cnephasia virgaureana*	Poland	A, L	F, S, L	Lipa et al. 1977
Thysanoptera	Aeolothripidae	*Melanthrips fuscus*	Poland	A	F	Lipa et al. 1977
	Phlaeothripidae	*Haplothrips aculeatus*	Poland	A	O	Lipa et al. 1977
		*Haplothrips setiger*	Poland	A	F	Lipa et al. 1977
	Thripidae	*Dictyothrips betae*	Poland	A	O	Lipa et al. 1977
		*Frankliniella intonsa*	Poland	A	F	Lipa et al. 1977
		*Limothrips denticornis*	Poland	A	O	Lipa et al. 1977
		*Taeniothrips atratus*	Poland	A	F	Lipa et al. 1977
		*Taeniothrips vulgatissimus*	Poland	A	O	Lipa et al. 1977
		*Thrips angusticeps*	Poland	A, L	F, S, L	Lipa et al. 1977
		*Thrips fuscipennis*	Poland	A	F, S, L	Lipa et al. 1977
		*Thrips major*	Poland	A	F, S, L	Lipa et al. 1977
		*Thrips tabaci*	Poland & United States	A, L	F, S, L	Lipa et al. 1977, Smith et al. 2011

This table is updated and modified by Lipa et al. (1977).

^a^Current scientific names in parenthesis.

^b^Collections by Lipa et al. (1977) were made primarily from Poland but may also represent observations from Czechoslovakia, Germany, and Hungary.

^c^Life stages: A = adults, E = eggs, L = larvae, N = nymphs, P = pupae.

^d^Plant parts: S = stem, L = leaves, R = root, F = flower, O = occasional, SP = seedpod.

^e^ Lipa et al. (1977) listed larva instead of nymph for the immature stage of these hemimetabolous insects.

### Coleoptera

Flea beetles (*Phyllotreta* spp. (Coleoptera: Chrysomelidae)) are major pests of Brassicaceae crops (Warwick et al. 2002), and they have been reported feeding on leaves and flowers of pennycress in the field and in the laboratory (Lamb and Palaniswamy 1990, Soroka and Grenkow 2013). Plant death was observed in the Czech Republic for pennycress growing in the field and being attacked from emergence onward by adults of flea beetles (Štolcová 2005). Several studies have more closely examined flea beetle preferences and performance on pennycress compared to other plants. In laboratory choice tests with adults of *Phyllotreta  cruciferae* (Goeze), feeding on *B. napus*, *Sinapis alba* L. (Brassicales: Brassicaceae), *Sinapis pubescens* L. (Brassicales: Brassicaceae) and *Crambe glabrata* DC. (Brassicales: Brassicaceae) was higher than on pennycress (Soroka and Grenkow 2013). Nonpreference resistance (i.e., antixenosis) of pennycress and *S. alba* has been observed for *P. cruciferae* (Gavloski et al. 2000). Choice tests done using leaf discs indicated that *Phyllotreta armoraciae* (Koch) prefers black mustard (*Brassica nigra* (L.) Koch (Brassicales: Brassicaceae)) and *S. alba* over pennycress (Nielsen et al. 1979), and that *Phyllotreta nemorum* (L.) and *P. cruciferae* prefer black mustard, radish (*Raphanus sativus* L. (Brassicales: Brassicaceae)), *S. alba*, and *B. napus* over pennycress (Nielsen et al. 2001). Similarly, *P. cruciferae* adults were found to exhibit a strong nonpreference response to pennycress compared to *Brassica juncea* (L.) Czernajew (Brassicales: Brassicaceae), *Brassica carinata* A. Braun (Brassicales: Brassicaceae), *B. napus*, *B. rapa*, and *S. alba* in Manitoba, Canada (Palaniswamy et al. 1997); and pennycress was only marginally acceptable to *Phyllotreta striolata* (F.) in Alberta, Canada (Meisner and Mitchell 1983). In no-choice conditions, survival of *P. cruciferae* adults on intact leaves of pennycress was as low as 9%, and adults that did survive on pennycress lost weight (Palaniswamy et al. 1997). In contrast, based on feeding experiments performed in the Czech Republic, Štolcová (2009) concluded that *Phyllotreta nigripes* (F.) and *Phyllotreta atra* (F.) do not show clear preferences among pennycress, wild mustard and *B. napus*. Overall, glucosinolates have been suggested as important factors for flea beetle host selection, and reducing the expression of glucosinolates in plants may change their susceptibility to being attacked by flea beetles (Soroka and Grenkow 2013).

Adults of cabbage seedpod weevil (*Ceutorhynchus obstrictus* (Marsham) (Coleoptera: Curculionidae)) were found on pennycress and other Brassicaceae weeds in Alberta, Canada (Dosdall and Moisey 2004). In a mixed stand of pennycress, wild mustard (*Sinapis arvensis* L. (Brassicales: Brassicaceae)) and flixweed (*Descurainia sophia* (L.) Webb ex Prantl (Brassicales: Brassicaceae)), *C. obstrictus* adults were found more frequently on wild mustard, and more on buds than stems or leaves independently of plant species (Dosdall and Moisey 2004). Pennycress did not appear to support *C. obstrictus* larval development as none of the pennycress seed pods had larval exit holes (Dosdall and Moisey 2004). Thus, pennycress may serve as an alternative food source for *C. obstrictus* adults in early spring until canola crops develop inflorescences that attract adults for feeding and reproduction.

Alfalfa weevil (*Hypera postica* (Gyllenhal) (Coleoptera: Curculionidae)) was found to oviposit on pennycress growing as a weed in alfalfa (*Medicago sativa* L. (Fabales: Fabaceae)) fields in Idaho, United States (Saad and Bishop 1969). After rearing the alfalfa plants in the laboratory, eggs of *H. postica* were observed to hatch, but larvae did not feed on pennycress, suggesting that *H. postica* larvae may migrate from pennycress to alfalfa in the field (Saad and Bishop 1969).

The sap beetles (*Meligethes aeneus* F. (currently *Brassicogethes aeneus* (F.)) and *M. viridescens* F. (currently *Brassicogethes viridescens* (F.)) (Coleoptera: Nitidulidae)) are pests of Brassicaceae crops in Europe (Williams 2010), and have been reported from pennycress (Lipa et al. 1977). However, *M. aeneus* and *M. viridescens* were not found on pennycress in a field study looking at the temporal distribution and dynamics of these species across a variety of Brassicaceae crops and weeds in May–July in Estonia (Metspalu et al. 2011). Metspalu et al. (2011) suggested that the apparent unsuitability of pennycress to *M. aeneus* and *M. viridescens* may be due to plant chemistry (e.g., glucosinolates or other secondary metabolites).

### Diptera

The serpentine leaf miner (*Liriomyza brassicae* (Riley) (Diptera: Agromyzidae)) was found to create mines on leaves of pennycress, yellow rocket (*Barbarea vulgaris* W.T. Aiton (Brassicales: Brassicaceae)) and black mustard growing in the wild in Wisconsin, United States, and in the laboratory with multiple lineages of *L. brassicae* (Tavormina 1982). The development time of multiple lineages of *L. brassicae* from oviposition to adults on pennycress was, on average, between 21.3 and 21.6 days (Tavormina 1982).

Some root maggot flies (Diptera: Anthomyiidae) are pests of germinating seeds and seedlings of crops. Cabbage root fly (*Erioischia brassicae* (Bouche) (currently *Delia radicum* (L.)) (Diptera: Anthomyiidae)) pupae were found in the soil near pennycress (up to an average of 0.9 pupae per plant) in a field survey of Brassicaceae weeds in the United Kingdom (Finch and Ackley 1977). However, when pennycress plants were inoculated with *E. brassicae* eggs in a greenhouse experiment, only 2% of the eggs produced pupae; the resulting pupae were smaller than those reared from other plants, and pennycress roots appeared healthy (i.e., not damaged by larval feeding) (Finch and Ackley 1977). In addition, pennycress is a reported host for *Delia floralis* (Fallén) (Diptera: Anthomyiidae) (Strickland 1938, Savage et al. 2016). Furthermore, adults of seed corn maggot (*Delia platura* (Meigen) (Diptera: Anthomyiidae)) have been found to be abundant on pennycress flowers (Forcella et al. 2023a). However, the potential impacts of the feeding of *D. platura* larvae on pennycress seeds or seedlings remain to be evaluated.

Swede midge (*Contarinia nasturtii* (Kieffer) (Diptera: Cecidomyiidae)) has been observed to feed on various Brassicaceae weeds including pennycress (Stokes 1953a, 1953b), but it was not found on pennycress growing in and near Brassicaceae vegetable fields in New York, United States (Chen et al. 2009). In a no-choice experiment, *C. nasturtii* oviposited on pennycress, but the number of larvae produced on pennycress was about 4 times lower than on cauliflower (Chen et al. 2009). In an ovipositional choice test experiment with pennycress, cauliflower, and 5 other Brassicaceae weeds, no *C. nasturtii* larvae were found on pennycress (Chen et al. 2009).

### Hemiptera

Aphids have been observed infesting pennycress in Wisconsin and Missouri, United States, and concern has been raised about their pest status for this new crop (Basnet and Ellison 2024). Cabbage aphid (*Brevicoryne brassicae* (L.) (Hemiptera: Aphididae)) was found to feed on pennycress in a laboratory experiment in Poland (Gabryś and Pawluk 1999). Results of tests using an electrical penetration graph showed that pennycress is an acceptable host for *B. brassicae* but that the phloem elements of pennycress may contain a deterrent factor (Gabryś and Pawluk 1999).

Turnip aphid (*Hyadaphis pseudobrassicae* (Davis) (currently *Lipaphis pseudobrassicae* (Davis)) (Hemiptera: Aphididae)) colonized and fed on pennycress in a series of greenhouse experiments in Iowa, United States (Jarvis 1970). Survival of pennycress plants 3–4 weeks after infestation with *H. pseudobrassicae* was 66% with no individual plants of pennycress showing resistance to this aphid (Jarvis 1970). In a second experiment, colonization of pennycress seedlings by alate *H. pseudobrassicae* was about 1/5 that of the average colonization on several susceptible genotypes of *B. napus*. In a third experiment, the population growth of *H. pseudobrassicae* on pennycress was about 1/4 that of the average population growth on several susceptible genotypes of *B. napus* (Jarvis 1970).

The plant bugs (*Lygus lineolaris* (Palisot), *Lygus elisus* Van Duzee, and *Lygus borealis* (Kelton) (Hemiptera: Miridae)) were found on pennycress growing in the wild in Manitoba, Canada (Gerber and Wise 1995). Overwintered adults of *L. lineolaris* appeared on pennycress during the second and third weeks of May, followed by nymphs in the third and fourth weeks of May (Gerber and Wise 1995). *Lygus* spp. nymphs remained abundant on pennycress from late May to late June and likely consisted mainly of *L. lineolarius* (Gerber and Wise 1995). However, by the time pennycress ripened at the end of June, few *Lygus* spp. remained on the plants, suggesting that this plant stage is unsuitable for *Lygus* spp. (Gerber and Wise 1995).

Two stink bugs (*Dolycoris baccarum* (L.) and *Carpocoris fuscispinus* (Boheman) (Hemiptera: Pentatomidae)) fed on and oviposited on pennycress in a laboratory study in Poland (Modnicki et al. 2019). Compared to 3 other Brassicaceae species (i.e., *Berteroa incana* (L.), *Diplotaxis tenuifolia* (L.), and flixweed), pennycress had the most feeding sites (i.e., punctures) from *C. fuscipinus* adults and the fewest from *D. baccarum* adults (Modnicki et al. 2019). Over a period of 3 days, *D. baccarum* oviposited, on average, the fewest eggs on pennycress (6.8 eggs), while *C. fuscipinus* oviposited an intermediate number of eggs on pennycress (13.8 eggs) compared to the other plants. The duration of egg development and rate of egg hatching for *D. baccarum* and *C. fuscipinus* did not differ significantly among host plants (Modnicki et al. 2019). On average, the duration of egg development of *D. baccarum* and *C. fuscipinus* on pennycress was 10.2 and 11.4 days, respectively (Modnicki et al. 2019). The average rate of egg hatching on pennycress for *D. baccarum* and *C. fuscipinus* was 60.3% and 58.6%, respectively (Modnicki et al. 2019).

### Lepidoptera

Bertha armyworm (*Mamestra configurata* Walker (Lepidoptera: Noctuidae)) survival and development were examined in a laboratory experiment in Canada with neonate first instars placed on excised leaf tissue of 10 plant species from 5 families, but *M. configurata* did not survive on pennycress (Dosdall and Ulmer 2004).

Southern armyworm (*Prodenia eridania* (Cramer) (currently *Spodoptera eridania* (Stoll)) (Lepidoptera: Noctuidae)) larvae were evaluated in the laboratory for feeding and growth on 74 plant species from multiple families in Illinois, United States (Soo Hoo and Fraenkel 1966). Larvae fed on pennycress, but pennycress did not support growth of *S. eridania* (Soo Hoo and Fraenkel 1966).

Cabbage looper (*Trichoplusia ni* (Hübner) (Lepidoptera: Noctuidae)) was found to feed on pennycress, broccoli, and 7 other agricultural weeds in a series of experiments with greenhouse-grown plants in British Columbia, Canada (Cameron et al. 2007). In a choice test experiment, adults of *T. ni* showed a preference to oviposit and feed on pennycress compared to broccoli (Cameron et al. 2007). However, in no-choice experiments, first instar *T. ni* consumed relatively little leaf tissue of pennycress, and less than 2% of those larvae survived after 7 days, whereas survival was greater than 60% on the other plant species (Cameron et al. 2007). Thus, Cameron et al. (2007) suggested that pennycress could be explored as a dead-end trap crop for *T. ni* management.

*Pieris* butterflies (Lepidoptera: Pieridae) have shown variable responses to pennycress. Multiple species, including *Pieris napi macdonnoughii* Remington (currently *Pieris macdunnoughi* Remington), *Pieris occidentalis* (Reakirt), *Pieris rapae* (L.) and *Pieris napi* (L.), were observed to oviposit on pennycress in natural plant communities or under laboratory conditions (Chew 1975, 1977, 1980, Slansky and Feeny 1977, Nakajima et al. 2013, Okamura et al. 2016). However, the larvae of *P. macdunnoughi* and *P. occidentalis* seem incapable of surviving when fed exclusively on pennycress (Chew 1975, 1980, Nakajima et al. 2013). Furthermore, the population fitness cost associated with oviposition of *P. macdunnoughi* on pennycress was estimated as 3% in natural landscapes, but no significant differences in population dynamics were observed over a 40-year period (Nakajima et al. 2013).

The larval growth of *P. rapae* when reared on pennycress was intermediate among multiple Brassicaceae plants in laboratory studies (Slansky and Feeny 1977, Okamura et al. 2016). Females of *P. napi* were found to lay eggs on pennycress plants of all sizes in Sweden, but more eggs were laid on smaller (e.g., 5 cm) compared to larger (e.g., 20 cm) plants, and smaller plants seemed to be more advantageous to survival and development of *P. napi* (Forsberg 1987).

Diamondback moth (*Plutella maculipennis* (Curt.) (currently *Plutella xylostella* (L.)) (Lepidoptera: Plutellidae)), which migrates to northern areas each spring, is found on pennycress in early spring in Ontario, Canada, and North Dakota, United States (Harcourt 1957, Kmec and Weiss 1997). Pennycress is a host for the first generation of *P. xylostella* prior to emergence of cultivated Brassicaceae plants (Harcourt 1957, Kmec and Weiss 1997). Adult *P. xylostella* in North Dakota were present from May to June in pennycress adjacent to field crops (Kmec and Weiss 1997). The density of *P. xylostella* eggs on leaves of pennycress varied from about 10–60 eggs m^−2^, and egg density was correlated with counts of adult *P. xylostella* in the field (Kmec and Weiss 1997). In Michigan, Idris and Grafius (1996) found intermediate levels of oviposition, development, and survival of *P. xylostella* on pennycress compared to several Brassicaceae plants, but similar egg hatches were observed. The average number of eggs per female *P. xylostella* on pennycress was lower than broccoli but similar to canola and 3 other weeds in a choice test and similar to broccoli, cauliflower, canola, and 6 other weeds in a no-choice test (Idris and Grafius 1996). Developmental time (from egg hatch to pupation) on pennycress (~13–14 days) was similar to red cabbage and several weeds (Idris and Grafius 1996). Furthermore, survival of *P. xylostella* larvae from hatching through the second instar was ~40% on pennycress, which was similar to red cabbage but lower than broccoli, kale, cauliflower, green cabbage, and canola (Idris and Grafius 1996). Survival of *P. xylostella* larvae from the third through fourth instar was high on pennycress (~85%) and similar to broccoli, kale, cauliflower, canola, and 2 weeds (Idris and Grafius 1996). The suitability of pennycress and 7 other Brassicaceae weeds as hosts for *P. xylostella* was examined in China using clip cages on intact leaves (Niu et al. 2014). Development time from egg to adult emergence (20.1 days) was the longest, the pupal weight (females: 4.3 mg; males: 3.1 mg) and survival from egg to adult (48.8%) were the lowest, and female adult longevity (15.1 days) and fecundity (192.4 eggs per female) were intermediate on pennycress compared to the other plants (Niu et al. 2014). Overall, *P. xylostella* raised on pennycress had the lowest intrinsic and finite rates of increase, longest generation time, and lowest net reproductive rate compared to the other plants (Niu et al. 2014).

Furthermore, pennycress and 3 other flowering plants were evaluated as nectar sources for adult *P. xylostella* in a laboratory experiment in Alberta, Canada (Munir et al. 2018). The longevity of adult *P. xylostella* (~25 days) on floral resources of pennycress was similar to that of *S. arvensis* and *B. napus* and higher than *Lobularia maritima* (L.) Desvaux (Brassicales: Brassicaceae), 10% honey solution, or water; however, the weight of *P. xylostella* adults was lower on floral resources of pennycress compared to *L. maritima*, 10% honey solution, or water (Munir et al. 2018).

Eri silkmoth (*Samia cynthia ricini* Boisduval (currently *Samia ricini* Anderson) (Lepidoptera: Saturniidae)) larval growth was examined in a greenhouse experiment in Japan using 12 Brassicaceae species varying in defensive traits, but first instar *S. cynthia ricini* fed on pennycress did not gain as much weight as those feeding on other species such as *Capsella bursa-pastoris* (L.) Medikus (Brassicales: Brassicaceae) or *Cardamine scutata* Thunberg (Brassicales: Brassicaceae) (Okamura et al. 2016).

### Thysanoptera

The onion thrips (*Thrips tabaci* Lindeman (Thysanoptera: Thripidae)) was found on pennycress in a field survey of 69 weed species near onion (*Allium cepa* L. (Asparagales: Amaryllidaceae)) fields in New York, United States (Smith et al. 2011). *Thrips tabaci* was found to reproduce on pennycress, and the highest *T. tabaci* abundance was observed on pennycress along with *B. vulgaris*, *S. arvensis*, and *Raphanus raphanistrum* L. (Brassicales: Brassicaceae) (Smith et al. 2011).

## Pennycress Response to Stresses Similar to Insect Injury

The response of pennycress to insect feeding is poorly understood. Some insight into how pennycress might respond could be inferred from how related crops (e.g., rapeseed and canola) respond to insect pests (e.g., Lamb 1989). Furthermore, a more specific understanding of how pennycress responds to insect feeding may be gained by examining the limited research on the response of pennycress to simulated injury (e.g., stand loss, defoliation, and apical stem injury).

Reductions in plant density due to insect feeding can have varying effects on crop yield, depending on the plasticity of the plant’s growth (Bardner and Fletcher 1974). A greenhouse experiment was performed in the United Kingdom to examine the response of pennycress to varying initial plant density and subsequent removal of neighboring plants at different timings (Matthies 1990). Pennycress seed was sown in pots and thinned after germination to targeted densities ranging from 1 to 64 seedlings per 12 cm × 12 cm pot (Matthies 1990). Then, for the thinning treatments, all neighboring plants were removed from around a central focal plant in each pot during the vegetative, flowing, and seed pod stages of pennycress (Matthies 1990). In this study, pennycress showed plasticity in response to plant density by producing more seeds per plant and larger seeds as plant density decreased (Matthies 1990). In addition, pennycress showed a greater ability to compensate when the removal of neighboring plants occurred at earlier growth stages (Matthies 1990).

Defoliation can have diverse physiological and morphological consequences on plants that can take place across a broad spectrum of timeframes (Clement et al. 1978, Hammond and Pedigo 1982, Aldea et al. 2005, van Klink et al. 2015). In a greenhouse experiment carried out in the United Kingdom, the effects of defoliation during 2 stages of pennycress flower development were investigated (Pyke 1989). For the defoliation treatments, all leaves on the primary stems of plants were removed when the first flower opened (“early defoliation”) or when 50% of flowers had lost their corolla and began enlarging (“mid-defoliation”) (Pyke 1989). Early defoliation caused fewer pods on defoliated plants compared to undefoliated plants (Pyke 1989). In contrast, mid-defoliation had no effect on the number of pods but resulted in a greater number of aborted seeds per pod on defoliated plants compared to control plants (Pyke 1989). Pyke (1989) noted that pods with aborted seeds were smaller but remained green on the plants and, therefore, may act as a photosynthetic source for seeds developing in other pods.

Apical injury to stems of plants that produce terminal flowers may result in increased branching from axillary buds and thereby increase the number of flowers but also results in loss of resources in the removed tissue, loss of photosynthetic area, and delays in seed production (Trumble et al. 1993, Fay et al. 1996). In a greenhouse study carried out in Indiana, United States, the impact of apical stem removal and fertilization on the branching and seed production of pennycress was assessed (Benner 1988). Early stem removal was performed when plants started to show internode elongation, and late stem removal was performed when most plants had formed flower buds or open flowers (Benner 1988). When the plants started to show internode elongation 20 days after being transplanted, early treatments involving apex removal and nutrient addition were conducted (Benner 1988). Late treatments, which also involved apex removal and nutrient addition, were performed 30 days after transplanting when most plants had formed flower buds or open flowers, which could potentially affect their response differently from plants that had not yet produced flowering structures (Benner 1988). Removal of the plant apex led to a decrease in the number of primary branches and an increase in the number of secondary and tertiary branches (Benner 1988). These effects were generally more pronounced when the apex was removed earlier than later (Benner 1988). Moreover, the number of fruits produced on all types of branches was higher in the plants where the apex was removed, regardless of the timing, with the early removed plants producing more fruits on these branches than the late-removal ones (Benner 1988). The total weight of seeds per plant was greater for early removal plants compared to late-removal plants, and the total weight of seeds for the control plants was generally greater than that of plants with either removal timing (Benner 1988). Benner (1988) observed that pennycress had some ability to compensate for apical removal by increasing the number of seeds produced but not the size of the seeds.

## Beneficial Insects Associated with Pennycress

Natural enemies such as parasitoids and predators could be affected by the incorporation of pennycress into cropping systems. Conflicting results have been observed for the effects of pennycress on *Diadegma insulare* (Cresson) (Hymenoptera: Ichneumonidae), a parasitoid of *P. xylostella*. In a field study in Michigan, United States, the longevity and fecundity of adult *D. insulare* females provided floral resources of pennycress was similar to (or slightly higher) than those provided only water and lower than those provided floral resources of *B. vulgaris* or *C. bursa-pastoris*, or a honey-water solution (Idris and Grafius 1995). In contrast, in a laboratory experiment in Alberta, Canada, the longevity of adult *D. insulare* on floral resources of pennycress (~25 days) was higher than on floral resources of *S. arvensis*, *B. napus*, or *L. maritima*, a honey-water solution, or water; however, the weight of *D. insulare* adults was lower on pennycress compared to *S. arvensis*, but higher than on water (Munir et al. 2018). In addition, indirect host-mediated effects of pennycress on *D. insulare* have also been examined. In a laboratory experiment in Michigan, United States, parasitism rates were lower and development times were longer for *D. insulare* when their host (i.e., *P. xylostella*) was reared on pennycress compared to several cultivated *Brassica* spp. (Idris and Grafius 1996).

Lipa et al. (1977) documented 9 species of predators associated with pennycress in Europe, including *Adonia variegata* (Goeze) (currently *Hippodamia variegata* (Goeze)), *Coccinella quatuordecimpustulata* (L.), *Coccinella quinquepunctata* (L.), *Coccinella septempunctata* L., *Coccinella undecimpunctata* (L.), *Propylaea quatuordecimpunctata* (L.) (Coloptera: Coccinellidae), *Tachyporus hypnorum* F. (Coloptera: Staphylinidae), *Nabis pseudoferus* Rem. (Hemiptera: Nabidae), and *Aeolothrips intermedius* Bagn. (Thysanoptera: Aeolothripidae). In the Midwest United States, Forcella et al. (2023a) found that adult Syrphidae were common visitors to pennycress flowers; however, the abundance of the predatory larvae of Syrphidae on pennycress was not quantified. In addition, the incorporation of pennycress into crop rotations may favor predatory insects. In a field experiment in Germany, a double-cropping system of pennycress and corn resulted in higher species richness and abundance of predatory ground beetles and higher species richness and diversity of spiders compared to 2 or 3 other common cropping systems (Groeneveld and Klein 2015a, Groeneveld et al. 2015b).

The early flowering time of pennycress can provide a source of early season floral resources to pollinators when most plants have not yet bloomed (Evangelista et al. 2017). The communities of pollinating insects associated with pennycress have received considerable attention (Mulligan and Kevan 1973, Groeneveld and Klein 2014, Eberle et al. 2015, Hassall et al. 2017, Stavert et al. 2018, Thom et al. 2018, Forcella et al. 2021, 2023a, 2023b).

## Conclusions

Pennycress has the potential to provide multiple benefits as a cover and oilseed crop in corn-soybean rotations in the Midwest United States. Significant advances have been made with genetic improvements and agronomic practices for this crop. However, as this crop begins to be adopted by farmers, there is an urgent need for guidance on potential pests, impacts of those pests, and management strategies.

To our knowledge, insect herbivore communities associated with pennycress grown as a crop or cover crop have not been fully documented. To provide a foundation for understanding potential insect pest threats, the scattered knowledge about insects associated with pennycress was compiled in this review. Among the taxa found associated with pennycress, several taxa, including *Phyllotreta* spp., Aphididae, *Lygus* spp. and *P. xylostella*, may pose the greatest pest threats to pennycress. However, because the primary focus of those studies was on pennycress growing in the wild (natural systems) or as a weed in agricultural systems, further work is needed to characterize the insect herbivore communities associated with pennycress growing under monoculture or intercropping scenarios representing how this crop will be deployed on the agricultural landscape. For example, seed maggots (Diptera: Anthomyiidae) are attracted to the flowers of pennycress (Forcella et al. 2023a), but it remains unknown if this might lead to increased abundance of such pests attacking seed of crops relay cropped into pennycress. Furthermore, as the domestication of pennycress continues, the susceptibility of new pennycress genotypes to insect pests will need to be evaluated.

Finally, we are not aware of any published reports regarding how pennycress responds to injury from insects. However, limited studies showing responses of pennycress (including yield loss or compensation) to reduction in plant density, and artificial defoliation and stem injury provide some insight as to how it might respond to insect pests. Further work is needed to examine the response of pennycress to actual and simulated insect injury. Such information will provide a foundation for the development of management guidelines such as economic injury levels and economic thresholds.

## References

[CIT0001] Aldea M , Hamilton JG, Resti JP, et al. 2005. Indirect effects of insect herbivory on leaf gas exchange in soybean. Plant Cell Environ. 28(3):402–411. 10.1111/j.1365-3040.2005.01279.x

[CIT0002] Alford DV , Nilsson C, Ulber B. 2003. Insect pests of oilseed rape crops. In: Alford DV, editor. Biocontrol of oilseed rape pests. 1st ed. Massachusetts: Wiley; p. 9–42.

[CIT0003] Bardner R , Fletcher KE. 1974. Insect infestations and their effects on the growth and yield of field crops: a review. Bull. Entomol. Res. 64(1):141–160. 10.1017/s0007485300027061

[CIT0004] Basnet P , Ellison S. 2024. Pennycress domestication and improvement efforts. Crop Sci. 64(2):535–559. 10.1002/csc2.21183

[CIT0005] Benner BL. 1988. Effects of apex removal and nutrient supplementation on branching and seed production in *Thlaspi arvense* (Brassicaceae). Am. J. Bot. 75(5):645–651. 10.1002/j.1537-2197.1988.tb13487.x 30139088

[CIT0006] Best KF , McIntyre GI. 1975. The biology of Canadian weeds: 9. *Thlaspi arvense* L. Can. J. Plant Sci. 55(1):279–292. 10.4141/cjps75-039

[CIT0007] Cameron JH , Isman MB, Upadhyaya MK. 2007. *Trichoplusia ni* growth and preference on broccoli and eight common agricultural weeds. Can. J. Plant Sci. 87(2):413–421. 10.4141/p06-032

[CIT0008] Cecchin A , Pourhashem G, Gesch RW, et al. 2021. Environmental trade-offs of relay-cropping winter cover crops with soybean in a maize-soybean cropping system. Agric. Sys. 189:103062. 10.1016/j.agsy.2021.103062

[CIT0009] Chen YH , Welter SC. 2005. Crop domestication disrupts a native tritrophic interaction associated with the sunflower, *Helianthus annuus* (Asterales: Asteraceae). Ecol. Entomol. 30(6):673–683. 10.1111/j.0307-6946.2005.00737.x

[CIT0010] Chen M , Shelton AM, Wang P, et al. 2009. Occurrence of the new invasive insect *Contarinia nasturtii* (Diptera: Cecidomyiidae) on cruciferous weeds. J. Econ. Entomol. 102(1):115–120. 10.1603/029.102.0116 19253625

[CIT0011] Chen C , Biere A, Gols R, et al. 2018. Responses of insect herbivores and their food plants to wind exposure and the importance of predation risk. J. Anim. Ecol. 87(4):1046–1057. 10.1111/1365-2656.12835 29672852

[CIT0012] Chew FS. 1975. Coevolution of pierid butterflies and their cruciferous foodplants. Oecologia 20(2):117–127. 10.1007/BF00369024 28308818

[CIT0013] Chew FS. 1977. Coevolution of pierid butterflies and their cruciferous foodplants. ii. The distribution of eggs on potential foodplants. Int. J. Org. Evol. 31(3):568–579. 10.1111/j.1558-5646.1977.tb01045.x 28563490

[CIT0014] Chew FS. 1980. Foodplant preferences of *Pieris* caterpillars (Lepidoptera). Oecologia 46(3):347–353. 10.1007/BF00346263 28310043

[CIT0015] Chopra R , Johnson EB, Emenecker R, et al. 2020. Identification and stacking of crucial traits required for the domestication of pennycress. Nature Food. 1(1):84–91. 10.1038/s43016-019-0007-z

[CIT0016] Clement CR , Hopper MJ, Jones LHP, et al. 1978. The uptake of nitrate by *Lolium perenne* from flowing nutrient solution II. Effect of light, defoliation, and relationship of CO_2_ flux. J. Exp. Bot. 29(5):1173–1183. 10.1093/jxb/29.5.1173

[CIT0017] Cubins JA , Wells MS, Frels K, et al. 2019. Management of pennycress as a winter annual cash cover crop. A review. Agron. Sustain. Dev. 39(5):46. 10.1007/s13593-019-0592-0

[CIT0018] Dosdall LM , Moisey DWA. 2004. Developmental biology of the cabbage seedpod weevil, *Ceutorhynchus obstrictus* (Coleoptera: Curculionidae), in spring canola, *Brassica napus*, in western Canada. Ann. Entomol. Soc. Am. 97(3):458–465. 10.1603/0013-8746(2004)097[0458:dbotcs]2.0.co;2

[CIT0019] Dosdall LM , Ulmer BJ. 2004. Feeding, development, and oviposition of bertha armyworm (Lepidoptera: Noctuidae) on different host plant species. Environ. Entomol. 33(3):756–764. 10.1603/0046-225x-33.3.756

[CIT0020] Eberle CA , Thom MD, Nemec KT, et al. 2015. Using pennycress, camelina, and canola cash cover crops to provision pollinators. Ind. Crops Prod. 75:20–25. 10.1016/j.indcrop.2015.06.026

[CIT0021] Evangelista RL , Cermak SC, Hojilla-Evangelista MP, et al. 2017. Field pennycress: a new oilseed crop for the production of biofuels, lubricants, and high-quality proteins. In: Biresaw G, Mittal KL, editors. Surfactants in tribology. Boca Ratan (FL, USA): CRC Press; p. 369–400.

[CIT0022] Fay PA , Hartnett DC, Knapp AK. 1996. Plant tolerance of gall-insect attack and gall-insect performance. Ecology 77(2):521–534. 10.2307/2265627

[CIT0023] Finch S , Ackley CM. 1977. Cultivated and wild host plants supporting populations of the cabbage root fly. Ann. Appl. Biol. 85(1):13–22. 10.1111/j.1744-7348.1977.tb00626.x

[CIT0024] Forcella F , Patel S, Lenssen AW, et al. 2021. Weather and landscape influences on pollinator visitation of flowering winter oilseeds (field pennycress and winter camelina). J. Appl. Entomol. 145(4):286–294. 10.1111/jen.12854

[CIT0025] Forcella F , Petersen M, Perry WL, et al. 2023a. Flies associated with floral canopies of the new oilseed crop, pennycress, in the Midwestern U.S.A. Gt. Lakes Entomol. 56(3-4):154–165. 10.22543/0090-0222.2463

[CIT0026] Forcella F , Portman ZM, Wells SS, et al. 2023b. Abundance and diversity of bees visiting flowering pennycress, a new oilseed crop in the Midwestern USA. Gt. Lakes Entomol. 56(1):99–107. 10.22543/0090-0222.2450

[CIT0027] Forsberg J. 1987. Size discrimination among conspecific hostplants in two pierid butterflies; *Pieris napi* L. and *Pontia daplidice* L. Oecologia 72(1):52–57. 10.1007/BF00385044 28312896

[CIT0028] Gabryś B , Pawluk M. 1999. Acceptability of different species of Brassicaceae as hosts for the cabbage aphid. Entomol. Exp. Appl. 91(1):105–109. 10.1046/j.1570-7458.1999.00471.x

[CIT0029] Gavloski JE , Ekuere U, Keddie A, et al. 2000. Identification and evaluation of flea beetle (*Phyllotreta cruciferae*) resistance within Brassicaceae. Can. J. Plant Sci. 80(4):881–887. 10.4141/p99-164

[CIT0030] Gerber GH , Wise IL. 1995. Seasonal occurrence and number of generations of *Lygus lineolaris* and *L. borealis* (Heteroptera: Miridae) in southern Manitoba. Can. Entomol. 127(4):543–559. 10.4039/ent127543-4

[CIT0031] Groeneveld JH , Klein A. 2014. Pollination of two oil‐producing plant species: camelina (*Camelina sativa* L. Crantz) and pennycress (*Thlaspi arvense* L.) double‐cropping in Germany. GCB Bioenergy 6(3):242–251. 10.1111/gcbb.12122

[CIT0032] Groeneveld JH , Klein A-M. 2015a. Pennycress-corn double-cropping increases ground beetle diversity. Biomass Bioenergy 77:16–25. 10.1016/j.biombioe.2015.03.018

[CIT0033] Groeneveld JH , Lührs HP, Klein AM. 2015b. Pennycress double-cropping does not negatively impact spider diversity. Agric. For. Entomol. 17(3):247–257. 10.1111/afe.12100

[CIT0034] Hammond RB , Pedigo LP. 1982. Determination of yield loss relationships for two soybean defoliators by using simulated insect defoliation techniques. J. Econ. Entomol. 75(1):102–107. 10.1093/jee/75.1.102

[CIT0035] Harcourt DG. 1957. Biology of the diamondback moth, *Plutella maculipennis* (Curt.) (Lepidoptera: Plutellidae), in eastern Ontario. II. Life-history, behaviour, and host relationships. Can. Entomol. 89(12):554–564. 10.4039/ent89554-12

[CIT0036] Hassall C , Owen J, Gilbert F. 2017. Phenological shifts in hoverflies (Diptera: Syrphidae): linking measurement and mechanism. Ecography 40(7):853–863. 10.1111/ecog.02623

[CIT0037] Hojilla-Evangelista MP , Evangelista RL, Isbell TA, et al. 2013. Effects of cold-pressing and seed cooking on functional properties of protein in pennycress (*Thlaspi arvense* L.) seed and press cakes. Ind. Crops Prod. 45:223–229. 10.1016/j.indcrop.2012.12.026

[CIT0038] Idris AB , Grafius E. 1995. Wildflowers as nectar sources for *Diadegma insulare* (Hymenoptera: Ichneumonidae), a parasitoid of diamondback moth (Lepidoptera: Yponomeutidae). Environ. Entomol. 24(6):1726–1735. 10.1093/ee/24.6.1726

[CIT0039] Idris AB , Grafius E. 1996. Effects of wild and cultivated host plants on oviposition, survival, and development of diamondback moth (Lepidoptera: Plutellidae) and its parasitoid *Diadegma insulare* (Hymenoptera: Ichneumonidae). Environ. Entomol. 25(4):825–833. 10.1093/ee/25.4.825

[CIT0040] Jarvis JL. 1970. Relative injury to some cruciferous oilseeds by the turnip aphid. J. Econ. Entomol. 63(5):1498–1502. 10.1093/jee/63.5.1498

[CIT0041] Johnson GA , Wells MS, Anderson K, et al. 2017. Yield tradeoffs and nitrogen between pennycress, camelina, and soybean in relay- and double-crop systems. Agron. J. 109(5):2128–2135. 10.2134/agronj2017.02.0065

[CIT0042] Kmec P , Weiss MJ. 1997. Seasonal abundance of diamondback moth (Lepidoptera: Yponomeutidae) on *Crambe abyssinica*. Environ. Entomol. 26(3):483–488. 10.1093/ee/26.3.483

[CIT0043] Lamb RJ. 1989. Entomology of oilseed brassica crops. Annu. Rev. Entomol. 34(1):211–229. 10.1146/annurev.en.34.010189.001235

[CIT0044] Lamb RJ , Palaniswamy P. 1990. Host discrimination by a crucifer-feeding flea beetle, *Phyllotreta striolata* (F.) (Coleoptera: Chrysomelidae). Can. Entomol. 122(5):817–824. 10.4039/ent122817-9

[CIT0045] Lipa JJ , Studziński A, Małachowska D. Insects and mites associated with cultivated and weedy cruciferous plants (Cruciferae) in Poland and central Europe. Warsaw, Poland: Polish Scientific Publishers; 1977. p. 354.

[CIT0046] Matthies D. 1990. Plasticity of reproductive components at different stages of development in the annual plant *Thlaspi arvense* L. Oecologia 83(1):105–116. 10.1007/BF00324641 28313250

[CIT0047] Meisner J , Mitchell BK. 1983. Phagodeterrency induced by two cruciferous plants in adults of the flea beetle *Phyllotreta striolata* (Coleoptera: Chrysomelidae). Can. Entomol. 115(9):1209–1214. 10.4039/ent1151209-9

[CIT0048] Metspalu L , Williams IH, Profile S, et al. 2011. Distribution of *Meligethes aeneus* (F.) and *M. viridescens* (F.) on cruciferous plants. Zemdirbyste-Agric 98(1):27–34.

[CIT0049] Mitich LW. 1996. Field Pennycress (*Thlaspi arvense* L.)—the stinkweed. Weed Technol. 10(3):675–678. 10.1017/s0890037x00040604

[CIT0050] Modnicki D , Lamparski R, Balcerek M, et al. 2019. The effect of the produce of phenolic compounds in some Brassicaceae plants on *Dolycoris baccarum* and *Carpocoris fuscispinus* feeding and development (Hemiptera: Pentatomidae). Acta Sci. Pol. Agric. 18(1):29–37. DOI: 10.37660/aspagr.2019.18.1.3

[CIT0051] Mohammed YA , Matthees HL, Gesch RW, et al. 2020. Establishing winter annual cover crops by interseeding into maize and soybean. Agron. J. 112(2):719–732. 10.1002/agj2.20062

[CIT0052] Moore SA , Wells MS, Gesch RW, et al. 2020. Pennycress as a cash cover-crop: improving the sustainability of sweet corn production systems. Agronomy 10(5):614. 10.3390/agronomy10050614

[CIT0053] Mulligan GA , Kevan PG. 1973. Color, brightness, and other floral characteristics attracting insects to the blossoms of some Canadian weeds. Can. J. Bot. 51(10):1939–1952. 10.1139/b73-248

[CIT0054] Munir S , Dosdall LM, Keddie A. 2018. Selective effects of floral food sources and honey on life‐history traits of a pest–parasitoid system. Entomol. Exp. Appl. 166(6):500–507. 10.1111/eea.12695

[CIT0055] Nakajima M , Boggs CL, Bailey S, et al. 2013. Fitness costs of butterfly oviposition on a lethal non-native plant in a mixed native and non-native plant community. Oecologia 172(3):823–832. 10.1007/s00442-012-2537-z 23254756

[CIT0056] Nielsen JK , Dalgaard L, Larsen LM, et al. 1979. Host plant selection of the horse‐radish flea beetle *Phyllotreta armoraciae* (Coleoptera: Chrysomelidae): feeding responses to glucosinolates from several crucifers. Entomol. Exp. Appl. 25(3):227–239. 10.1111/j.1570-7458.1979.tb02875.x

[CIT0057] Nielsen JK , Hansen ML, Agerbirk N, et al. 2001. Responses of the flea beetles *Phyllotreta nemorum* and *P. cruciferae* to metabolically engineered *Arabidopsis thaliana* with an altered glucosinolate profile. Chemoecology 11(2):75–83. 10.1007/pl00001835

[CIT0058] Niu YQ , Sun YX, Liu TX. 2014. Development and reproductive potential of diamondback moth (Lepidoptera: Plutellidae) on selected wild crucifer species. Environ. Entomol. 43(1):69–74. 10.1603/EN13206 24367918

[CIT0059] Okamura Y , Sawada Y, Hirai MY, et al. 2016. Effects of different secondary metabolite profiles in plant defense syndromes on specialist and generalist herbivores. Entomol. Sci. 19(2):97–103. 10.1111/ens.12172

[CIT0060] Palaniswamy P , Lamb RJ, Bodnaryk RP. 1997. Antibiosis of preferred and non-preferred host-plants for the flea beetle, *Phyllotreta cruciferae* (Goeze) (Coleoptera: Chrysomelidae). Can. Entomol. 129(1):43–49. 10.4039/ent12943-1

[CIT0061] Pyke DA. 1989. Limited resources and reproductive constraints in annuals. Funct. Ecol. 3(2):221. 10.2307/2389304

[CIT0062] Saad A , Bishop G. 1969. Egg-laying by the alfalfa weevil in weeds. J. Econ. Entomol. 62(5):1226–1227.

[CIT0063] Savage J , Fortier A-M, Fournier F, et al. 2016. Identification of *Delia* pest species (Diptera: Anthomyiidae) in cultivated crucifers and other vegetable crops in Canada. Can. J. Arthropod Identif. 29(29):1–40.

[CIT0064] Sedbrook JC , Phippen WB, Marks MD. 2014. New approaches to facilitate rapid domestication of a wild plant to an oilseed crop: example pennycress (*Thlaspi arvense* L.). Plant Sci. 227:122–132. 10.1016/j.plantsci.2014.07.008 25219314

[CIT0065] Selling GW , Hojilla-Evangelista MP, Evangelista RL, et al. 2013. Extraction of proteins from pennycress seeds and press cake. Ind. Crops Prod. 41(1):113–119. 10.1016/j.indcrop.2012.04.009

[CIT0066] Slansky F , Feeny P. 1977. Stabilization of the rate of nitrogen accumulation by larvae of the cabbage butterfly on wild and cultivated food plants. Ecol. Monogr. 47(2):209–228. 10.2307/1942617

[CIT0067] Smith EA , Ditommaso A, Fuchs M, et al. 2011. Weed hosts for onion thrips (Thysanoptera: Thripidae) and their potential role in the epidemiology of iris yellow spot virus in an onion ecosystem. Environ. Entomol. 40(2):194–203. 10.1603/en10246

[CIT0068] Soo Hoo CF , Fraenkel G. 1966. The selection of food plants in a polyphagous insect, *Prodenia eridania* (Cramer). J. Insect Physiol. 12(6):693–709. 10.1016/0022-1910(66)90115-6

[CIT0069] Soroka J , Grenkow L. 2013. Susceptibility of Brassicaceous plants to feeding by flea beetles, *Phyllotreta* spp. (Coleoptera: Chrysomelidae). J. Econ. Entomol. 106(6):2557–2567. 10.1603/ec13102 24498758

[CIT0070] Stavert JR , Pattemore DE, Bartomeus I, et al. 2018. Exotic flies maintain pollination services as native pollinators decline with agricultural expansion. J. Appl. Ecol. 55(4):1737–1746. 10.1111/1365-2664.13103

[CIT0071] Stokes BM. 1953a. Biological investigations into the validity of *Contarinia* species living on the Cruciferae, with special reference to the swede midge, *Contarinia nasturtii* (Kieffer). Ann. Appl. Biol. 40(4):726–741. 10.1111/j.1744-7348.1953.tb01110.x

[CIT0072] Stokes BM. 1953b. The host plant range of the Swede midge (*Contarinia nasturtii* Kieffer) with special reference to types of plant damage. Tijdschrift Over Plantenziekten 59(3):82–90. 10.1007/bf02106324

[CIT0073] Štolcová J. 2005. Insect injury and mortality of seedlings of field pennycress (*Thlaspi arvense* L.). Plant Prot. Sci. 41(1):21–26. 10.17221/2736-pps

[CIT0074] Štolcová J. 2009. Feeding preferences of *Phyllotreta* herbivores to winter rape and chosen weeds. Plant Prot. Sci. 45(4):156–160. 10.17221/40/2008-pps

[CIT0075] Strickland EH. 1938. An annotated list of the Diptera (flies) of Alberta. Can. J. Res. 16d(7):175–219. 10.1139/cjr38d-012 20278189

[CIT0076] Tavormina SJ. 1982. Sympatric genetic divergence in the leaf-mining insect *Liriomyza brassicae* (Diptera: Agromyzidae). Evolution Int. J. Org Evolution 36(3):523–534. 10.1111/j.1558-5646.1982.tb05073.x 28568038

[CIT0077] Thom MD , Eberle CA, Forcella F, et al. 2018. Specialty oilseed crops provide an abundant source of pollen for pollinators and beneficial insects. J. Appl. Entomol. 142(1-2):211–222. 10.1111/jen.12401

[CIT0078] Trumble JT , Kolodny-Hirsch DM, Ting IP. 1993. Plant compensation for arthropod herbivory. Annu. Rev. Entomol. 38(1):93–119. 10.1146/annurev.en.38.010193.000521

[CIT0079] van Klink R , Schrama M, Nolte S, et al. 2015. Defoliation and soil compaction jointly drive large-herbivore grazing effects on plants and soil arthropods on clay soil. Ecosystems 18(4):671–685. 10.1007/s10021-015-9855-z

[CIT0080] Vaughn SF , Isbell TA, Weisleder D, et al. 2005. Biofumigant compounds released by field pennycress (*Thlaspi arvense*) seedmeal. J. Chem. Ecol. 31(1):167–177. 10.1007/s10886-005-0982-4 15839488

[CIT0081] Warwick SI , Francis A, Susko DJ. 2002. The biology of Canadian weeds. 9. *Thlaspi arvense* L. Can. J. Plant Sci. 82(4):803–823. 10.4141/p01-159

[CIT0082] Williams IH. Biocontrol-based integrated management of oilseed rape pests. 1st ed. Netherlands: Springer Dordrecht; 2010. p. 1–461. 10.1007/978-90-481-3983-5

[CIT0083] Zheng X , Koopmann B, Ulber B, et al. 2020. A global survey on diseases and pests in oilseed rape—current challenges and innovative strategies of control. Front. Agron. 2:590908. 10.3389/fagro.2020.590908

